# Institutional change and Institutional Integration in France: Health Regional Agencies what consequences for the development of integrated services delivery?

**Published:** 2012-09-04

**Authors:** Hélène Trouvé, Olivier Saint-Jean, Dominique Somme

**Affiliations:** Engineer of Research, National Foundation of Gerontology, Paris, France; Professor of Geriatric, Georges Pompidou European Hospital, University of Paris Descartes, Paris, France; Geriatrician, Georges Pompidou European Hospital, University of Paris Descartes, Paris, France

**Keywords:** integrated networks, institutional integration, institutional change, Social healthcare system

## Abstract

**Purpose:**

In France, the development of integrated services delivery for elderly (ISDE) has been hindered by the fragmentation of the decision-making institutions. By merging the healthcare and medicosocial institutions, the Health Regional Agencies (HRA), responsible for developing ISDE, theoretically constitutes a solution. The study examines how the HRA received and implemented the legislative mandate of the development of the ISDE.

**Theory:**

We used the model of “receptivity to change strategic” developed by A.M. Pettigrew in the context of reforms of the National Health Service (NHS).

**Method:**

An empirical study was conducted in 2011 in two phases: 1) 10 case studies of HRA, 2) questionnaire sent to all 26 HRAs.

**Results and conclusion:**

The concentration of the decision powers in HRAs favors the deployment of ISDE. Three considerations limit this consideration: 1) lack of legislative instruments allows the HRA to reproduce a fragmented internal organization; 2) lack of prerogative on the social institutions; these institutions can develop contradictory policies; 3) new definition of norms and governance may slow down the deployment of ISDE.

**Discussion:**

The deployment of ISDE relies on regional institutions that are subjected to changes. The on-going analysis will determine whether the new frames of reference and governance comply with the principles of services integration.

Powerpoint presentation available at http://www.integratedcare.org at congresses – San Marino – programme.

